# A Challenging Case of Eyelid Ptosis and Diplopia Induced by Pembrolizumab

**DOI:** 10.7759/cureus.28330

**Published:** 2022-08-24

**Authors:** Daniela Garcez, Ana Isabel Clara, Maria Francisca Moraes-Fontes, José Bravo Marques

**Affiliations:** 1 Neuro-Oncology, Champalimaud Foundation, Lisbon, PRT; 2 Digestive Unit, Champalimaud Foundation, Lisbon, PRT; 3 Internal Medicine, Champalimaud Foundation, Lisbon, PRT

**Keywords:** immunotherapy adverse effect, myocarditis, myasthenia gravis, myositis, ocular myositis, pembrolizumab, immune checkpoint inhibitors

## Abstract

We present the case of an 83-year-old female patient with gastric adenocarcinoma, who developed a subacute onset of eyelid ptosis and ophthalmoparesis, while being treated with pembrolizumab, raising the suspicion of myasthenia gravis. Workup exposed a broader systemic involvement, with liver, cardiac and skeletal muscle being affected as well. Further investigation lead us to change our initial diagnosis to multisystem inflammatory syndrome with isolated symptomatic ocular myositis, induced by pembrolizumab. High-dose steroids and immunoglobulin were started with a good outcome.

## Introduction

Immune checkpoint inhibitors (ICIs) revolutionized the treatment of various malignancies. However, with their wide use, immune-related adverse events (irAEs) have been reported, involving multiple body organs, including both central and peripheral nervous systems. An incidence of 2-4% neurological irAEs has been reported, which albeit infrequent but still can be serious and life-threatening [1]. They most commonly involve the peripheral nerve, muscle end-plate, and muscle. Several parts of the nervous system can be simultaneously involved and can occur together with non-neurological irAEs in other organs [2]. Their early recognition and proper handling are of paramount importance for therapeutic success.

## Case presentation

**Figure 1:**
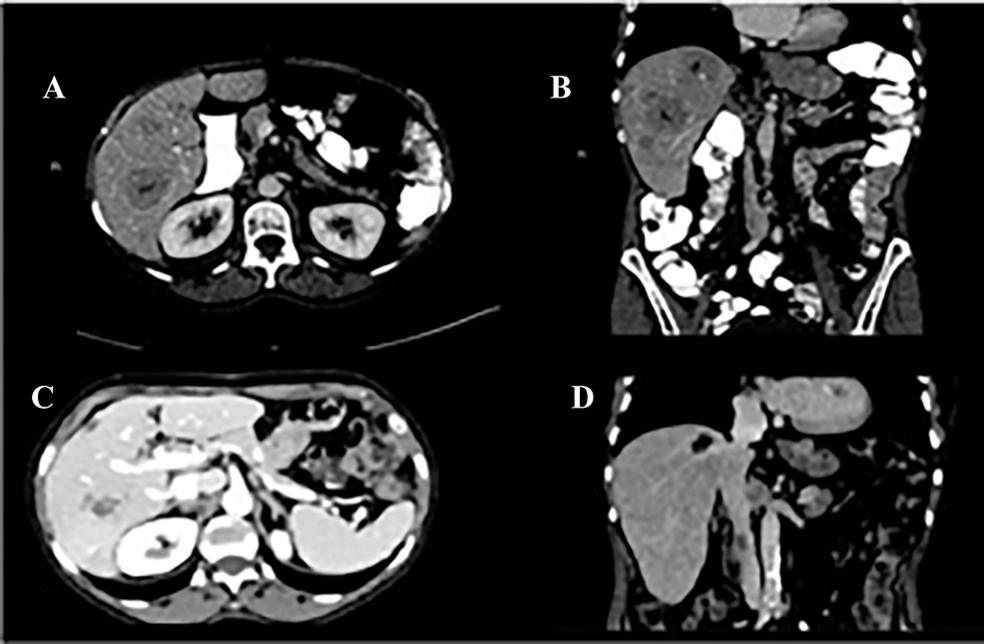
Abdominal CT before (A, axial view; B, sagittal view) and after (C, axial view; D, sagittal view) four months of treatment with pembrolizumab

An 83-year-old female patient, a former high-school teacher, was diagnosed with adenocarcinoma of gastroesophageal junction (while asymptomatic) in May 2021, in the context of routine blood tests (GGT, 164 U/L; <38 U/L). Her past medical history was unremarkable except for a partial gastrectomy at age of 13 years due to congenital pyloric stenosis. Staging examinations showed bi-lobar multiple liver metastases and loco-regional adenopathies. Due to the presence of programmed death-ligand 1 (PD-L1) expression in the tumor (=10) and older age, she was started on pembrolizumab (2 mg/kg every three weeks). She received four cycles with good tolerance and good clinical and radiological response (Figures 1A-1D).

Before cycle five, an increase in liver enzymes was detected (aspartate transaminase {AST}, 45 U/L {<34}; alanine aminotransferase {ALT}, 72 U/L {10-49}, gamma-glutamyl transferase {GGT} 116 U/L {<38}) and an immune-oncology consult was requested. During the consultation, the patient mentioned having blurred vision and difficulty opening her eyes for the past five days. She also felt more tired than usual and had a sense of imbalance while walking. Though symptoms were present along all day, she used to feel better in the morning. On suspicion of myasthenia gravis, a neurology consult was sought. On examination, she had bilateral symmetric eyelid ptosis, bilateral external ophthalmoparesis with binocular horizontal diplopia, normal strength of orbicularis oculi, and a positive Cogan sign. There was no dysarthria or dysphagia, and muscle strength was normal, with no inducible fatigability on repetitive movements. Muscle stretch reflexes, including ankle jerk reflexes, were increased, with no Babinski sign. She had no limb ataxia, but was unable to walk a straight line; superficial and deep sensations were normal. Cognitive testing was normal, as was her general physical examination, including respiratory status.

The subacute onset of bilateral ptosis and external ophthalmoparesis, without any symptoms or signs of facial and limb weakness or sensory abnormalities, suggested an involvement of the peripheral nervous system, specifically ocular motor nerves, neuromuscular junction or eyelid, and ocular muscles. However, because of mild gait imbalance and increased reflexes, we could not exclude involvement of the central nervous system.

The patient's medical history excluded hereditary, occupational and traumatic etiologies. Stroke, neurodegenerative causes, and infection were also unlikely due to the subacute onset of symptoms, and the absence of fever and meningeal signs. A toxic-metabolic cause or leptomeningeal tumor dissemination jeopardizing eyelid and ocular muscle nerves could not be excluded on clinical grounds alone. An intermediate phenotype between Miller Fisher syndrome and Bickerstaff brainstem encephalitis was also considered in the main differential diagnosis. However, an auto-immune adverse event induced by pembrolizumab seemed the most reasonable etiology, namely an ICI-induced myasthenia gravis or ocular myositis.

Blood tests identified an increased creatine kinase (CK) of 2336 UI/L (normal range: 34-145 U/L) and a troponin of 1922 ng/L (normal range: <60) in the absence of decreased muscle strength or pain and despite the lack of cardiac symptoms. ECG showed a left bundle branch block, already identified in previous ECGs. Transthoracic echocardiogram was normal.

**Figure 2:**
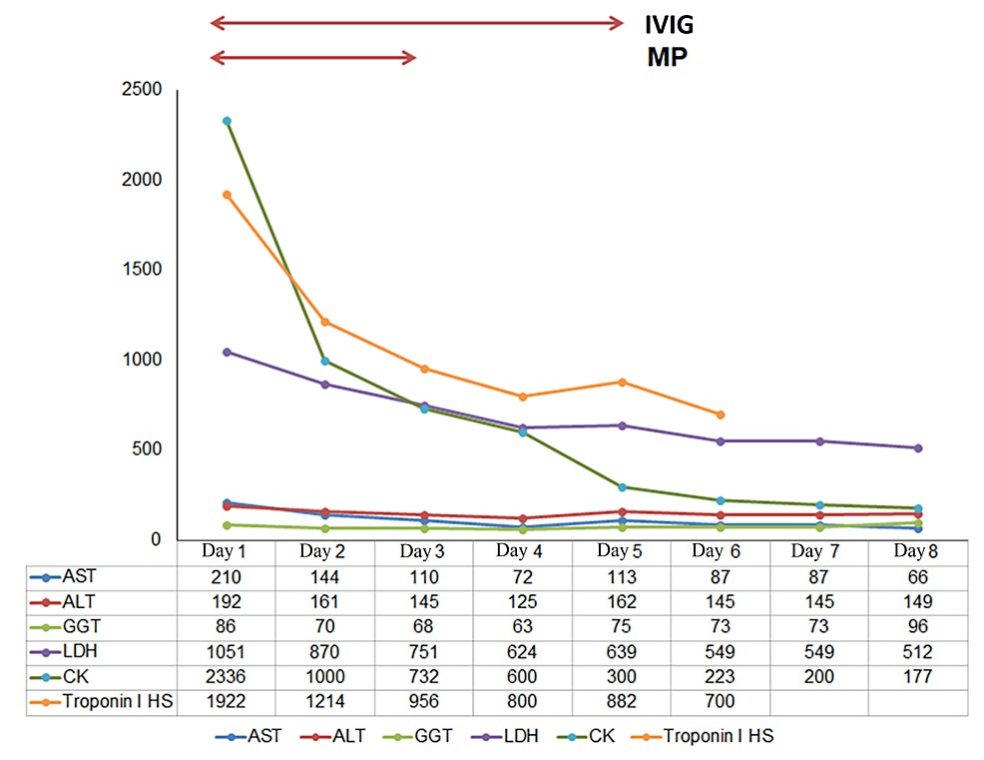
Biochemistry evolution after starting intravenous methylprednisolone and immunoglobulin AST: aspartate aminotransferase (U/L); ALT: alanine transaminase (U/L); GGT: gamma-glutamyl transferase (U/L); LDH: lactate dehydrogenase (U/L); troponin I HS: high-sensitivity troponin I (ug/L); CK: creatine kinase (U/L); IVIG: intravenous immunoglobulin; MP: methylprednisolone

On suspicion of a multisystem inflammatory syndrome induced by pembrolizumab with either myasthenia gravis or ocular myositis with occult systemic myositis, myocarditis, and hepatitis, the patient was hospitalized and started on high dose methylprednisolone (1 g/day during three days), followed by oral prednisolone (1 mg/kg during two weeks with weaning) plus intravenous immunoglobulin (0.4 mg/kg during five days). While in hospital, cardiac muscle, skeletal muscle, and hepatic enzymes improved and it became clear to us that the patient's symptoms did not fluctuate throughout the day (Figure 2).

**Figure 3:**
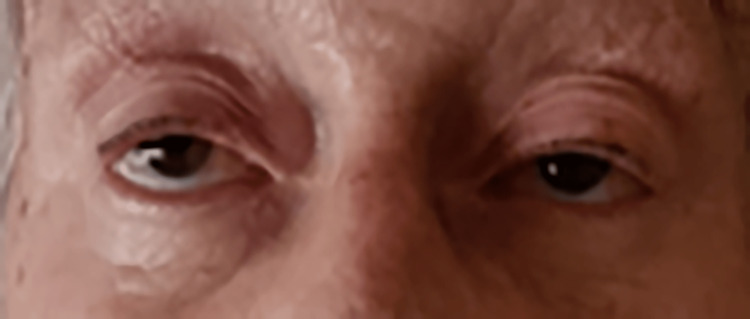
Picture taken at 7:35 a.m. after the patient wakes up, showing bilateral ptosis

Fatigability was also difficult to prompt throughout repetitive movements. Even after resting all night, the patient was still waking up with droopy eyes and double vision (Figure 3).

We tested the patient with intravenous neostigmine, without improvement of ptosis or ocular palsy and we did a trial with oral pyridostigmine after hospital discharge (60 mg three times a day during one week), also without improvement. Anti-acetylcholine receptor (anti-AChR) antibodies were negative. An electromyogram (EMG) was performed seven days after the patient was discharged from the hospital not taking pyridostigmine or prednisolone and being almost asymptomatic. No decrementing response to repetitive nerve stimulation was identified. Conduction studies and needle electromyography were also normal. A single-fiber EMG was not performed due to its lack of specificity for distinguishing myasthenia gravis from myositis. Brain MRI was normal, not suggesting Bickerstaff brainstem encephalitis, and cervical MRI showed cervical spondylotic myelopathy, probably the cause of her gait imbalance and hyperreflexia.

Our final diagnosis was probable systemic myositis with symptomatic ocular myositis, plus asymptomatic myocarditis and hepatitis, all related to treatment with pembrolizumab.

Six months after discharge, the patient had total disappearance of diplopia but residual eyelid ptosis remained. Her sense of imbalance also improved, though a slight walking unsteadiness persisted on examination, readily dismissed by the patient.

After discontinuation of pembrolizumab, the patient remained in remission without having any active oncological treatment. After five months of follow-up, tumor progression was identified on body CT. Chemotherapy with capecitabine was then started with good tolerance and response.

## Discussion

Ocular involvement frequently occurs in myasthenia gravis. It is rarely a part of systemic myositis, but as part of ICIs AE, systemic and ocular myositis can both occur without or with concomitant myasthenia gravis. Differentiating myasthenia gravis from ocular myositis can be difficult [3,4]. Although Cogan Sign is associated with myasthenia gravis, it is neither specific nor pathognomonic [5,6]. Other clues are the presence or absence of fluctuating symptoms, fatigability, serum muscle enzymes, presence or absence of serum myositis and myasthenia gravis antibodies, as well as findings on EMG and response to anticholinesterase drugs.

Our patient had external ophthalmoplegia, bilateral ptosis, involving the levator palpebrae superioris, with no orbicularis oculi weakness or significant daily fluctuations or inducible fatigability. CK and troponin levels were high; AChR and muscle-specific kinase (MuSK) antibodies were negative. Myositis autoantibodies (anti-Jo1, SRP, MDA-5, TIF-1- gamma, NXP2 antibodies) and anti-ganglioside antibodies (GM1, GM2, GM3, GD1a, GD1b, GD1b, GT1b, GQ1b) were also negative. No improvement was seen after intravenous neostigmine. Although performed after hospital discharge and with the patient clinically improved, a trial of oral pyridostigmine had no additional therapeutic effect on residual ptosis and diplopia. Electromyography, including repetitive nerve stimulation, as well as orbital MRI, was also normal. We could not find clinical and laboratory evidence to support the diagnosis of myasthenia gravis and findings suggest that our patient had asymptomatic generalized myositis and concurrent myocarditis with symptomatic isolated ocular myositis.

Idiopathic inflammatory myositis, involving levator palpebrae superioris and ocular muscles is rare, but when due to ICIs, it may preferentially involve these muscles [3]. Some authors advocate that predominant ocular involvement could be caused by the presence of specific autoantigens expressed in ocular muscles [4]. In fact, the occurrence of ICIs-related ocular myositis resembles ocular findings in patients with idiopathic eyelid ptosis and ophthalmoplegia, reinforcing the idea of a possible autoimmune etiology [4]. Myositis induced by ICIs is thought to be caused by activated CD8+ T lymphocytes which directly attack self-muscles [3].

Several cases of simultaneous myocarditis with myositis and/or myasthenia gravis due to ICIs have been reported in the literature. A recent systematic review by Pathak et al. reported a total of 60 patients, 22 with concomitant myositis with myocarditis plus myasthenia gravis and 38 patients who had myocarditis with myositis [7]. Among them, just one case suffered from gastrointestinal cancer, a 79-year-old man with a gastric adenocarcinoma under pembrolizumab, with a 10-day history of bilateral eyelid ptosis, diplopia, and bilateral external ophthalmoplegia, who died. In fact, 60% of 60 patients from this review died in hospital because of acute complications. All of them started treatment with steroids alone.

According to clinical practice guidelines in the management of ICIs adverse events, patients developing myositis and/or myasthenia gravis often present concomitantly with myocarditis, and workup for these concomitant toxicities should be strongly considered. These patients should be hospitalized because of their dismal prognosis [8]. However, to our knowledge, no guidance is given on how to approach treatment of overlapping syndromes as clinical practice orientations are focused on treating each disease individually.

Safa et al. reported a single center experience with ICI-related myasthenia gravis in 65 patients, with 37% (24 patients) experiencing concurrent myositis [9]. The authors suggest that IVIG or plasmapheresis (PLEX) should be used as a first-line regimen in myasthenia gravis, regardless of severity of initial symptoms, as they failed to be able to predict the course of the disease. They also reinforce that steroid use can cause an acute exacerbation of idiopathic myasthenia gravis symptoms and elimination of pathogenic antibodies from the serum, by using IVIG or PLEX, may more rapidly improve symptoms [9].

In our case, we used high-dose methylprednisolone and IVIG, as the initial treatment as myasthenia gravis was initially suspected, but not confirmed. The patient, who presented with overlapping irAEs involving skeletal and cardiac muscles, had a successful response to treatment and a stable outcome with no adverse events of treatment.

In the context of irAEs prompt diagnosis and confirmation of myasthenia gravis can be difficult and elusive and withholding treatment can be hazardous. Our case illustrates the efficacy of using stronger immunosuppression/modulation therapy, not only in patients with suspected myasthenia gravis but also with concurrent myositis and myocarditis, even when symptoms are minor.

Although ICI rechallenge after temporary discontinuation appears plausible in many cases [10], it is associated with a higher incidence of all-grade irAEs than initial treatment with ICIs (27.5-55%) [11]. In our patient, it was decided to discontinue ICI and start chemotherapy for tumor recurrence.

## Conclusions

The cornerstone of the treatment of neurological irAEs is early recognition. Although the clinical picture can apparently suggest a mild disease, we want to highlight the potential of ICIs inducing a multisystem inflammatory syndrome, with few or no symptoms in an early phase. On the other hand, the differential diagnosis can be challenging when solely relying on ocular symptoms and signs. Thus, a comprehensive workup should be performed. The main and initial treatment is with steroids, but additional or further immunosuppression should strongly be considered, specifically when an overlapping syndrome is under consideration.
